# Medication-related burden and its association with medication adherence among elderly tuberculosis patients in Guizhou, China: a cross-sectional study

**DOI:** 10.3389/fphar.2024.1416005

**Published:** 2024-08-29

**Authors:** Yun Wang, Sisi Jian, Zhongfeng Huang, Huijuan Chen, Yuanxia Hu, Shilin Fang

**Affiliations:** ^1^ Key Laboratory of Environmental Pollution Monitoring and Disease Control, Ministry of Education, School of Public Health, Guizhou Medical University, Guiyang, China; ^2^ Department of Tuberculosis, Guiyang Public Health Clinical Center, Guiyang, China; ^3^ Institute of Health Promotion and Education, Guizhou Center for Disease Prevention and Control, Guiyang, China; ^4^ School of Medicine and Health Management, Guizhou Medical University, Guiyang, China

**Keywords:** tuberculosis, medication-related burden, medication adherence, elderly, LMQ-3

## Abstract

**Introduction:**

Tuberculosis (TB) morbidity and mortality are significantly increasing in the elderly worldwide. Their optimal health outcomes are hampered by medication related burden (MRB) and poor treatment adherence. Understanding th e MRB status from patients’ perspectives and its association with adherence among elderly TB patients will help achieve the End TB targets. Thus, we aimed to identify the incidence of MRB and nonadherence among elderly TB patients in Guizhou, and determine their association.

**Methods:**

A cross-sectional study was conducted in three prefectures with high TB notifications in Guizhou in 2022. The data were collected via face-to-face structured interviews. MRB was measured using the Living with Medicines Questionnaire version 3 (LMQ-3), which consists of eight domains. Nonadherence was assessed by treatment interruption, which was defined as any interruption lasting at least 1 day at any time within the last 3 months. A binary unconditional logistic regression model was used to determine the association between variables.

**Results:**

Of the 405 elderly TB patients enrolled, 49.4% and 42.7% of the respondents perceived suffering from moderate and high MRB, respectively. The incidence of nonadherence among patients was 33.6%. Patients with higher scores in domain 2 (practical difficulties) [*OR*_adj_ = 1.19; 95% *CI* (1.11–1.28)] and domain 4 (side effects burden of prescribed medications) [*OR*_adj_ = 1.16; 95% *CI* (1.06–1.27)] were more likely to experience nonadherence. But, patients with higher scores in domain 8 (control/autonomy of medicine use) [*OR*_adj_ = 0.70; 95% CI (0.61, 0.81)] were more likely to occur adherence. Patients with a high education level [*OR*_adj_ = 0.29; 95% *CI* (0.08, 0.92)] had a decreased risk of nonadherence, but those with a living expense from a retirement salary [*OR*_adj_ = 2.55; 95% *CI* (1.16, 5.71)] had an increased risk of nonadherence.

**Discussion:**

The incidence of MRB and medication nonadherence is high among elderly TB patients in Guizhou. The significant associations between the three domains of MRB and nonadherence highlight that measuring MRB in multiple dimensions using the LMQ-3 in elderly TB patients could assist clinicians in providing patient-centered care, and multifaceted interventions targeting the identified problems should be implemented to reduce MRB and nonadherence among elderly TB patients in Guizhou.

## 1 Introduction

Tuberculosis (TB) is a chronic respiratory infectious disease caused by the *bacillus Mycobacterium tuberculosis* (World Health Organization, 2023a). TB is a serious threat to human health worldwide (World Health Organization, 2023b). In 2022, there were about 7.5 million newly diagnosed TB cases and 1.3 million deaths caused by TB globally (World Health Organization, 2023a). The largest number of new TB cases occurred in WHO’s South-East Asian Region (46%), followed by the African Region (23%) and the Western Pacific (18%). Around 87% of new TB cases occurred in the 30 high TB burden countries, with more than two-thirds of the global total in China, Bangladesh, Democratic Republic of the Congo, India, Indonesia, Nigeria, Pakistan and the Philippines. Over 80% of cases and deaths are in low- and middle-income countries (World Health Organization, 2023b). The TB epidemic is most prevalent among the elderly population, and the TB mortality rate is high (Caraux-Paz et al., 2021). Medication nonadherence remains one of the major factors increasing TB morbidity and mortality (Grigoryan et al., 2022). Long-term and multidrug anti-TB therapy leads to a heavy medication-related burden (MRB) (Chiang et al., 2023) and nonadherence (Kwon et al., 2022) in patients. Elderly TB patients may bear a heavier burden from taking multiple drugs due to decreased physical function and increased comorbidities (Chin et al., 2023). Thus, understanding the MRB status and its association with adherence among elderly TB patients can inform effective intervention efforts to improve TB cure rates and promote progress toward achieving the End TB targets (Talukdar et al., 2022).

Previous studies have reported MRB among TB patients from different perspectives, such as pill burden (Ting et al., 2020), social burden (e.g., travel restrictions due to medication) (Asriwati et al., 2021), burden of management (e.g., need for reminding patients to take medicine) (Hassani et al., 2023) and financial burden (e.g., costs of treatment) (Liu et al., 2020). However, there is a lack of studies on the comprehensive measurement of MRB in TB patients with a unified scale. Moreover, many published studies on the association between MRB and adherence in TB patients are qualitative studies. The Living with Medicines Questionnaire version 3 (LMQ-3) has been developed and validated from the perspective of patients (Katusiime et al., 2018; Krska et al., 2019). The LMQ-3 includes eight domains of potential burden on patients and quantifies the types of problems patients encounter when taking their medicines. The LMQ-3 provides a comprehensive assessment of MRB status in patients. Thus, it is currently used to measure MRB in the population with chronic noncommunicable diseases (Awad et al., 2020; Alqallaf et al., 2022) and some infectious diseases (Katusiime et al., 2023). Therefore, a quantitative study is essential to comprehensively measure MRB using the LMQ-3 and determine its association with adherence among TB patients.

China has the third highest burden of TB globally after India and Indonesia, with 560,847 newly diagnosed TB cases and 2205 deaths caused by TB in 2022 (World Health Organization, 2023a; National Bureau of Statistics of China, 2023). The elderly population (≥60 years) in China has exceeded 264 million, accounting for 18.7% of the total population (World Health Organization, 2023b). China is facing the challenges of the shifting prevalence of TB from younger to elderly individuals due to an aging population, longer life expectancy and disease reactivation (Cheng et al., 2020). A study pointed to the high incidence and risk of developing TB among elderly people in China, and the prevention and control of TB in elderly people need more attention (Cheng et al., 2020). Guizhou is a low-income province in China with a high TB incidence (Zhou et al., 2021). There are 9 prefectures and 88 counties with a population of 38.56 million (National Bureau of Statistics of China, 2023). From 2011 to 2020, the incidence of TB in elderly individuals in Guizhou was approximately twice that of the whole population (Ma et al., 2021). In this context, Guizhou is an appropriate place to conduct this study. The present study aimed to assess MRB status using the LMQ-3, identify the incidence of MRB and nonadherence among elderly TB patients in Guizhou, and to determine their associations.

## 2 Materials and methods

### 2.1 Study design, setting and population

A cross-sectional study was conducted in three prefectures (Guiyang, Bijie and Qiandongnan) with high TB notification in Guizhou Province (Cheng et al., 2020; Chen et al., 2021; Wang et al., 2022). The study population included outpatients visiting three designated TB treatment hospitals in each prefecture from April to July 2022. The inclusion criteria were as follows: 1) diagnosed with TB at 60 years of age or older, 2) received at least one medication prescribed for TB and any disease/condition, and 3) agreed to participate in this survey and signed informed consent forms. Patients who had severe mental disorders, hearing impairments or language communication disorders were excluded from the survey. All TB outpatients who met the inclusion criteria were included in this study and underwent a face-to-face questionnaire interview.

The sample size was determined by the formula $n=\frac{z_{1-\alpha /2}^{2}}{d^{2}}\times p\left(1-p\right)$ . The minimum sample size (391) was calculated based on a nonadherence rate (31.3%) with 95% confidence limits among TB patients in a cross-sectional survey (Natarajan et al., 2022). Finally, 405 TB patients who met the inclusion criteria were consecutively enrolled in the study with a nonprobability sampling technique.

### 2.2 Study questionnaire

The questionnaires were divided into four sections: demographic characteristics, mediation-related characteristics, nonadherence and MRB.

Section 1 describes the demographic characteristics of the participants, including gender, age (years), residence, source of living expenses, annual household income (Chinese Yuan), loss of productivity due to TB, debt due to TB, special subsidies, medical insurance type and education level (3 levels: 1. primary education or less refers to those who have completed or not completed 6 years of primary education. 2. secondary education refers to those who have completed 3-year junior high school and/or a 3-year senior high school. 3. high education refers to those who have obtained degrees, diplomas, or certificates offered by universities, colleges, and various professional schools).

Section 2 describes the mediation-related characteristics of the participants, including the type of TB, treatment classification of TB, comorbidities, need for support with using medicines, number of drug types and adverse drug reactions (ADRs). In Section 3, medication nonadherence is assessed by treatment interruption, which is defined as any interruption lasting at least 1 day at any time within the last 3 months (Bastard et al., 2015; Yin et al., 2018).

Section 4: MRB is assessed by the LMQ-3 scale. This scale consists of eight domains and 41 items. The eight domains are domain 1: relationships/communication with healthcare professionals about medicines (5 items); domain 2: practical difficulties (7 items); domain 3: cost-related burden (3 items); domain 4: side-effects burden of prescribed medications (4 items); domain 5: perceived effectiveness of medicines (6 items); domain 6: attitudes/concerns about medicine use (7 items); domain 7: interference with day-to-day life (6 items); and domain 8: control/autonomy of medicine use (3 items). All items in the LMQ-3 are scored on a five-point Likert scale (Katusiime et al., 2018). The scoring rules are coded as positive (from 1 = strongly agree to 5 = strongly disagree) or negative (from 5 = strongly agree to 1 = strongly disagree). Of the 41 items, 16 are coded positively (Items 3, 4, 7, 11, 13, 14, 15, 20, 24, 25, 26, 28, 32, 34, 39, 40), and 25 are coded negatively (Items 1, 2, 5, 6, 8, 9, 10, 12, 16, 17, 18, 19, 21, 22, 23, 27, 29, 30, 31, 33, 35, 36, 37, 38, 41). The total LMQ-3 score is the sum of the subtotal scores of the eight domains. The subtotal score for each domain is the sum of the scores for the items it contains. The average score for each domain is calculated by its subtotal score divided by the number of items it contains. MRB is categorized based on the total LMQ-3 score: 1) minimum burden (41–87); 2) moderate burden (88–110); and 3) high burden (≥111) (Alqallaf et al., 2022). The LMQ-3 demonstrated adequate construct validity and reliability (domain loadings ranging from 0.617-0.933 and a Cronbach’s α ranging from 0.714-0.932) (Katusiime et al., 2023).

### 2.3 Data management and analysis

Epidata software version 3.1 was utilized for data entry and storage. All statistical analyses were performed using R software version 4.3.1. The median and interquartile range (IQR) are used for continuous variables; otherwise, frequencies and percentages are used. Variables associated with medication adherence were explored initially by univariate analysis, including the Mann‒Whitney *U* test (for continuous variables), chi‒square test and Fisher’s exact probability method (for categorical variables). Multivariate analysis was performed in 4 steps: 1) The component plus residual plot was used to observe whether there were linear relationships between the scores in the eight domains of MRB and the logit-transformed values of medication adherence. If a linear relationship exists between them, then a logistic regression model can be used (Ma et al., 2023). (Supplementary Figure S1) 2) The Pearson correlation coefficients between the scores of the eight domains were estimated and are presented in a heat plot (Supplementary Figure S2). A Pearson correlation coefficient less than 0.8 indicated that there was no significant multicollinearity between them (Senaviratna and Cooray, 2019). 3) Variables with *p* < 0.15 in the univariate analysis were included in the binary unconditional logistic regression model. 4) The likelihood ratio test was assessed at each step and used to determine the final model where only variables with a *p* < 0.05 remained. The Hosmer–Lemeshow goodness of fit test was used to assess the fit of the multivariable binary logistic regression model. Significance was set at α = 0.05, and testing was two-sided.

Ethical clearance was obtained from Medical Ethics Committee of Guizhou Medical University (The approval number is No. 265 of 2021).

## 3 Results

### 3.1 Demographic and medication-related characteristics of the respondents

The demographic and medication-related characteristics of the respondents are listed in Table 1. A total of 405 respondents were included in the study. A total of 264 (65.2%) respondents were males. The majority (80.7%) of the respondents were aged 60–74 years. More than half (59.5%) of the respondents were rural dwellers, and only 34 (8.4%) respondents had a high education level. A total of 221 (54.6%) respondents obtained their main living expenses from their children, while 22 (5.4%) respondents obtained those from subsistence allowances. The annual household income of 236 (58.3%) respondents was less than 30,000 Chinese yuan. Forty-nine (12.1%) of the respondents lost productivity due to TB, and 44 (10.9%) had debt due to TB treatment. Sixty-nine (17.0%) respondents had special subsidies. Only 6 (1.5%) did not have any medical insurance. The number of drug-resistant TB (DR-TB) patients was 95 (23.5%). In addition, 223 (55.1%) of the respondents had other comorbidities. A total of 297 (73.3%) needed support using medicines. A total of 78 (19.3%) respondents used five or more drugs, and 322 (79.5%) experienced ADRs.

**TABLE 1:** Demographic and mediction-related characteristics of respondents (*n* = 405).

Characteristics	Frequency *n* (%)	Characteristics	Frequency *n* (%)
Demographic characteristics		Special subsidies	
Gender		Yes	69 (17.0)
Male	264 (65.2)	No	336 (83.0)
Female	141 (34.8)	Medical insurance type	
Age (Years)		URBMI	310 (76.5)
60–74	327 (80.7)	UEBMI	89 (22.0)
≥75	78 (19.3)	None	6 (1.5)
Residence		Mediction-related characteristics	
Rural	241 (59.5)	Type of TB	
Urban	164 (40.5)	TB	310 (76.5)
Education level		DR-TB	95 (23.5)
Primary education or less	236 (58.3)	Treatment classification of TB	
Secondary education	135 (33.3)	Initial treatment	310 (76.5)
High education	34 (8.4)	Re-treatment	95 (23.5)
Source of living expenses		Comorbidities	
Children	221 (54.6)	Yes	223 (55.1)
Retirement salary	103 (25.4)	No	182 (44.9)
Deposit	32 (7.9)	Need for support with using medicines	
Subsistence allowance	22 (5.4)	Yes	297 (73.3)
Others	27 (6.7)	No	108 (26.7)
Annual household income (Chinese Yuan)		Number of drug types	
<30,000	236 (58.3)	1–4	327 (80.7)
≥30,000	169 (41.7)	≥5	78 (19.3)
Loss productivity due to TB		ADRs	
Yes	49 (12.1)	Yes	322 (79.5)
No	356 (87.9)	No	83 (20.5)
Debt due to TB			
Yes	44 (10.9)		
No	361 (89.1)		

Abbreviations: TB, tuberculosis; URBMI, urban resident basic medical insurance; UEBMI, urban employee basic medical insurance; DR-TB, drug resistant tuberculosis; ADRs: adverse drug reactions.

### 3.2 Assessment of MRB using LMQ-3

**FIGURE 1:**
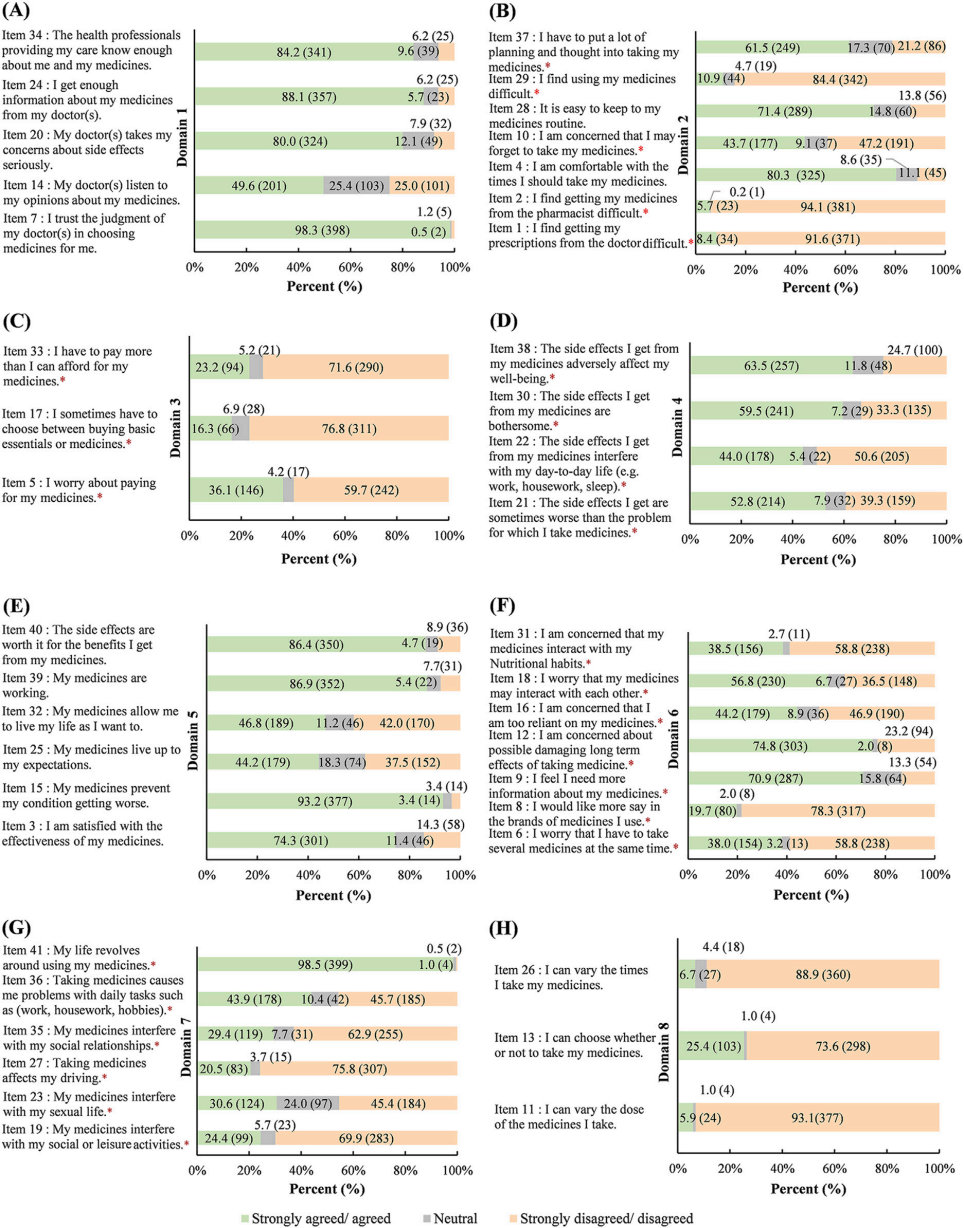
Responses to individual items in the eight domains of the LMQ-3 (*n* = 405), %(*n*) **(A)**: Domain 1 (Relationships/communication with healthcare professionals about medicines) **(B)**: domain 2 (Practical Difficulties) **(C)**: domain 3 (Cost-related burden) **(D)**: domain 4 (Side effects burden of prescribed medications) **(E)**: domain 5 (Perceived effectiveness of medicines) **(F)**: domain 6 (Attituddes/Concerns about medicine use) **(G)**: domain 7 (Interferences with day-to-day life) **(H)**: domain 8 (Control/Autonomy of medicine use). *Negatively worded items were reverse coded for calculation of the score. Abbreviations: LMQ-3, Living with Medicines Questionnaire version 3.

The responses of the respondents to individual items within the eight domains of the LMQ-3 are shown in Figure 1.

Domain 1 shows the relationships/communication with healthcare professionals about medicines and includes 5 items (Figure 1A). A total of 25.0% of respondents reported that doctors do not listen to their opinions on medicines (Item 14), and 7.9% reported that doctors do not care about their side effects (Item 20). A total of 6.2% of the respondents reported that they could not obtain enough information about their medicines from their doctors (Item 24) and did not care enough about medicines prescribed by health professionals (Item 34). A total of 1.2% replied of respondents reported that they do not trust the judgment of their doctors in choosing medicines for them (Item 7).

Domain 2 represents practical difficulties and includes 7 items (Figure 1B). The majority of respondents did not find it difficult to receive prescriptions (Item 1, 91.6%) or medicines (Item 2, 94.1%) from their doctors/pharmacists. Most of the respondents were comfortable taking medicines (Item 4, 80.3%) and easily maintained their medicine routine (Item 28, 71.4%). In terms of medicine use, 10.9% of respondents reported difficulty (Item 29), 43.7% reported that they may forget to take medicines (Item 10), and 61.5% reported planning and thinking about taking medicines (Item 37).

Domain 3 assesses cost-related burden and has 3 items (Figure 1C). A total of 36.1% of respondents worried about the cost of medicine (Item 5), 23.2% paid more than they could afford for their medicines (Item 33), and 16.3% had to choose between necessities and medicines (Item 17).

Domain 4 represents the side effect burden of prescribed medications and contains 4 items (Figure 1D). A total of 52.8% of the respondents replied that the side effects of the medicines were sometimes worse than the disease (Item 21). The side effects affected daily life for 44.0% of the respondents (Item 22), wellbeing for 63.5% of the respondents (Item 38), and bothered 59.5% of the respondents (Item 30).

Domain 5 represents the perceived effectiveness of medicines and contains 6 items (Figure 1E). A total of 74.3% of respondents were satisfied with the efficacy of medicines (Item 3) and 44.2% felt that the medicines met their expectations (Item 25). A total of 93.2% of the respondents reported that the medicines prevented their condition from worsening (Item 15), and 46.8% could live according to their own will by taking medicines (Item 32). A total of 86.9% reported that the medicines were working (Item 39), and 86.4% reported that the side effects were worthwhile if the medicines worked (Item 40).

Domain 6 represents attitudes/concerns about medicine use and contains 7 items (Figure 1F). A total of 19.7% wanted to choose the brands of medicines (Item 8), and most of the respondents wanted to get more information (Item 9, 70.9%) and learn about the long-term damage (Item 12, 74.8%) of the medicines. Some of the respondents were concerned about taking several medicines at the same time (Item 6, 38.0%), being overly reliant on medicines (Item 16, 44.2%), having interactions between medicines (Item 18, 56.8%) and having interactions between medications and nutritional habits (Item 31, 38.5%).

Domain 7 interferes with day-to-day life and has 6 items (Figure 1G). Some respondents reported that medicines interfered with their normal social or leisure activities (Item 19, 24.4%), sexual life (Item 23, 30.6%), driving (Item 27, 20.5%), social relationships (Item 35, 29.4%), and daily tasks (Item 36, 43.9%). A total of 98.5% reported that their life revolves around using their medicines (Item 41).

Domain 8 represented the control/autonomy of medicine use and had 3 items (Figure 1H). A total of 93.1% of respondents thought that they could not vary the dose of their medicines (Item 11), 88.9% reported that they could not vary the number of times they took their medicines (Item 26), and 73.6% responded that they could not choose whether or not to take medicines (Item 13).

**FIGURE 2:**
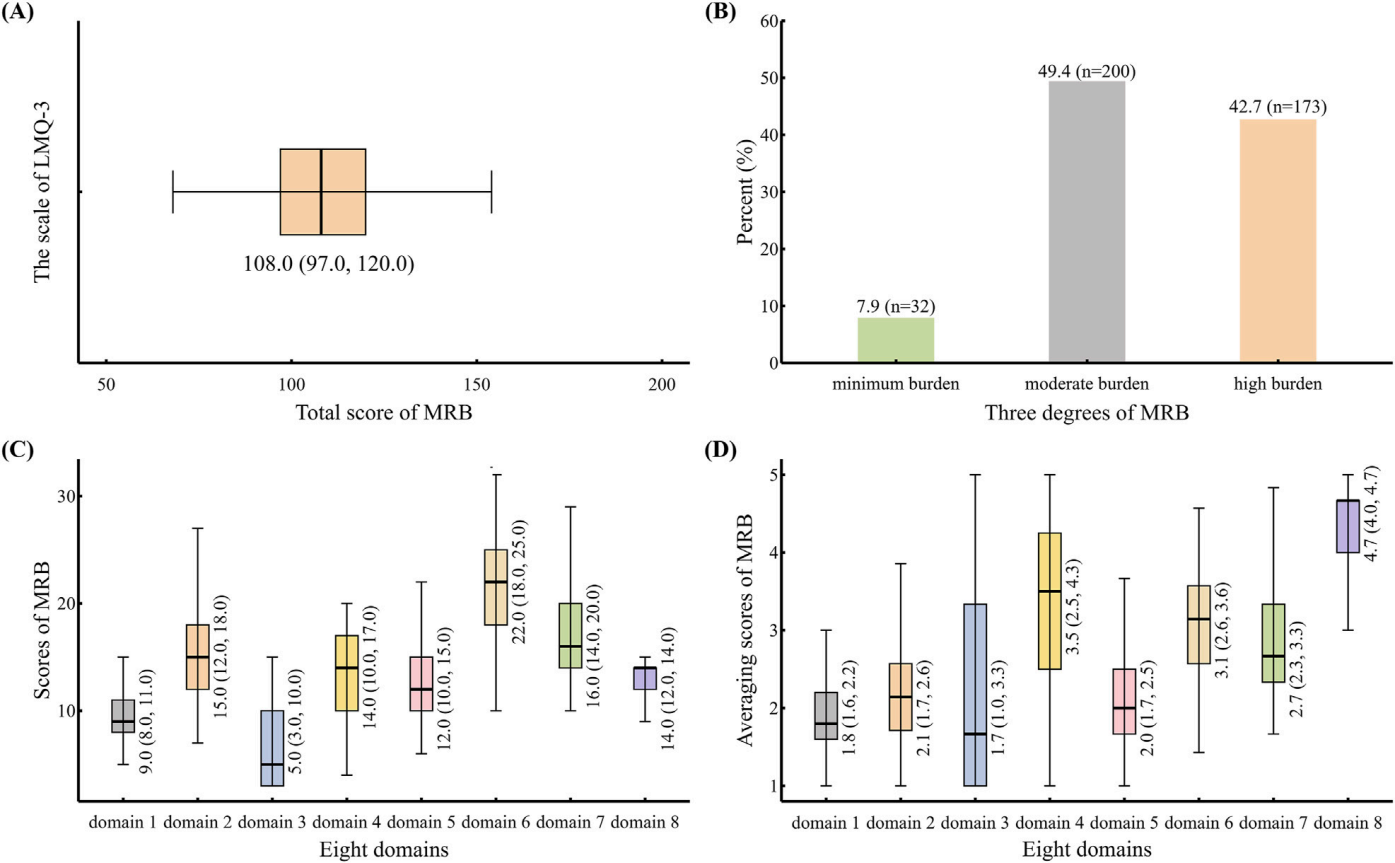
The scale of LMQ-3 was used to measure perceived MRB of respondents (n = 405) **(A)**: Distribution for median and IQR of total scores in the scale of LMQ-3 **(B)**: Percent of perceived different degrees of MRB in respondents **(C)**: Distribution of scores in the eight domains **(D)**: Distribution of averaging scores by the number of items contained in each domain. Abbreviations: LMQ-3, Living with Medicines Questionnaire version 3; IQR, interquartile range; MRB, medication-related burden.

The LMQ-3 was used to measure the respondents’ perceived MRB (*n* = 405), as shown in Figure 2. The median and IQR of the total scores were 108.0 (97.0, 120.0) (Figure 2A). Among 405 respondents, 49.4% (n = 200) and 42.7% (n = 173) perceived suffering from moderate and high degrees of MRB, respectively (Figure 2B). Of the eight domains, domain 6 had the highest score, with a median of 22.0 and an IQR (18.0, 25.0). The scores of domain 3 were the lowest, with a median of 5.0 and an IQR (3.0, 10.0) (Figure 2C). After averaging scores by the number of items contained in each domain, the average scores of domain 8 were the highest, with a median (4.7) and IQR (4.0, 4.7), followed by those of domain 4, with a median (3.5) and IQR (2.5, 4.3). The average score of domain 3 was still the lowest, with a median of 1.7 and an IQR (1.0, 3.3) (Figure 2D).

### 3.3 Associations between medication adherence and MRB and respondent characteristics according to univariate analysis

Associations of the respondents’ medication adherence with each domain and the respondents’ characteristics are given in Table 2, 3, respectively. Of the 405 respondents, 136 (33.6%) had medication nonadherence. Table 2 shows that scores across six domains (domains 1, 2, 3, 4, 5, and 8) were associated with respondents’ medication adherence (*p* < 0.05). Table 3 shows that treatment classification of the TB and ADRs were related to the medication adherence of the respondents (*p* < 0.05).

**TABLE 2:** Association between the eight domains of MRB and medication adherence (*n* = 405).

Variables	Medication		*u*	*p*
	Adherence	Nonadherence		
**Overall *n* (%)**	269 (66.4)	136 (33.6)		
Scores of eight domains* Median (IQR)				
Domain 1 (Relationships/communication with healthcare professionals about medicines)	9.0 (8.0, 11.0)	9.0 (8.0, 12.0)	−3.429	<0.001
Domain 2 (Practical Difficulties)	14.0 (11.0, 16.0)	17.0 (14.0, 21.0)	−7.449	<0.001
Domain 3 (Cost-related burden)	5.0 (3.0, 9.0)	6.0 (3.0, 11.0)	−2.008	0.045
Domain 4 (Side effects burden of prescribed medications)	12.0 (9.0, 15.0)	16.0 (12.0, 18.0)	−5.109	<0.001
Domain 5 (Perceived effectiveness of medicine)	11.0 (9.0, 14.0)	13.0 (10.0, 18.3)	−4.091	<0.001
Domain 6 (Attituddes/Concerns about medicine use)	22.0 (18.0, 25.0)	23.0 (19.0, 26.0)	−1.523	0.128
Domain 7 (Interferences with day-to-day life)	16.0 (14.0, 19.0)	17.0 (14.8, 20.0)	−1.807	0.078
Domain 8 (Control/Autonomy of medicine use)	14.0 (13.0, 15.0)	12.0 (9.0, 14.0)	7.140	<0.001

Note: *Higher scores indicating greater burden.

Abbreviations: MRB, medication-related burden; IQR, interquartile range.

**TABLE 3:** Association between respondents’characteristics and medication adherence (*n* = 405).

Characteristics	Medication		$\chi^{2}$	*p*
	Aderence *n* (%)	Nonadherence *n* (%)		
Demographic characteristics				
Gender				
Male	169 (64.0)	95 (36.0)	1.966	0.161
Female	100 (70.9)	41 (29.1)		
Age (Years)				
60–74	218 (66.7)	109 (33.3)	0.046	0.829
0 ≥ 75	51 (65.4)	27 (34.6)		
Residence				
Rural	154 (63.9)	87 (36.1)	1.694	0.193
Urban	115 (70.1)	49 (29.9)		
Education level				
Primary education or less	148 (62.7)	88 (37.3)	4.651	0.098
Secondary education	94 (69.6)	41 (30.4)		
High education	27 (79.4)	7 (20.6)		
Source of living expenses				
Children	151 (68.3)	70 (31.7)	7.923	0.094
Retirement salary	67 (65.0)	36 (35.0)		
Deposit	24 (75.0)	8 (25.0)		
Subsistence allowance	9 (40.9)	13 (59.1)		
Others	18 (66.7)	9 (33.3)		
Annual household income (Chinese Yuan)				
<30,000	156 (66.1)	80 (33.9)	0.026	0.873
≥30,000	113 (66.9)	56 (33.1)		
Loss productivity due to TB				
Yes	27 (55.1)	22 (44.9)	3.201	0.074
No	242 (68.0)	114 (32.0)		
Debt due to TB				
Yes	25 (56.8)	19 (43.2)	2.040	0.153
No	244 (67.6)	117 (32.4)		
Special subsidies				
Yes	42 (60.9)	27 (39.1)	1.149	0.284
No	227 (67.6)	109 (32.4)		
Medical insurance type				
URBMI	205 (66.1)	105 (33.9)	*	0.582
UEBMI	61 (68.5)	28 (31.5)		
None	3 (50.0)	3 (50.0)		
Mediction-related characteristics				
Type of TB				
TB	208 (67.1)	102 (32.9)	0.272	0.602
DR-TB	61 (64.2)	34 (35.8)		
Treatment classification of TB				
Initial treatment	215 (69.4)	95 (30.6)	5.104	0.024
Re-treatment	54 (56.8)	41 (43.2)		
Comorbidities				
Yes	149 (66.8)	74 (33.2)	0.035	0.852
No	120 (65.9)	62 (34.1)		
Need for support with using medicines				
Yes	197 (66.3)	100 (33.7)	0.004	0.949
No	72 (66.7)	36 (33.3)		
Number of drug types				
1–4	220 (67.3)	107 (32.7)	0.463	0.496
≥5	49 (62.8)	29 (37.2)		
ADRs				
Yes	203 (63.0)	119 (37.0)	8.030	0.005
No	66 (79.5)	17 (20.5)		

Note: *, It was caculated by Fisher’s exact probability method.

Abbreviations: TB, tuberculosis; URBMI, urban resident basic medical insurance; UEBMI, urban employee basic medical insurance; DR-TB, drug resistant tuberculosis; ADRs: adverse drug reactions.

### 3.4 Associations between MRB and medication adherence after adjusting for covariates

**FIGURE 3:**
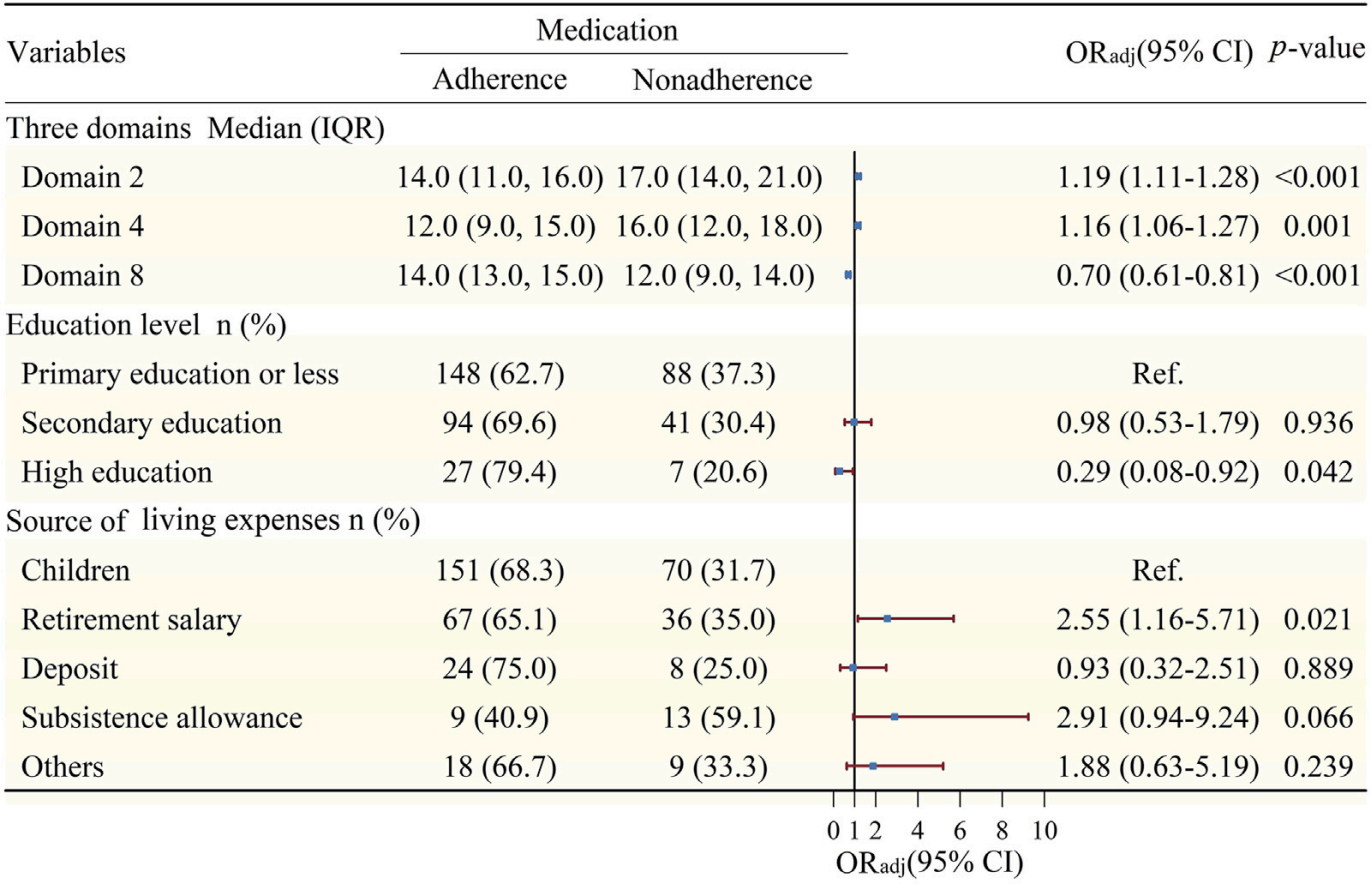
Association between MRB and medication adherence adjusting for covariates*. Notes: The Hosmer–Lemeshow goodness of fit test showed a *p*-value of 0.834 (>0.05); *Adjusted for education level, source of living expenses, loss productivity due to TB, Treatment classification of TB, ADRs. Abbreviations: MRB, medication-related bueden; *OR*_adj_, adjusted odds ratio; *CI*, confidence interval; Ref., reference group; TB, tuberculosis; ADRs, adverse drug reactions.

There were associations between three domains (domains 2, 4, and 8) and medication adherence (*p* < 0.05) according to the binary unconditional logistic regression shown in Figure 3 (full results for the associations between all relevant variables and medication adherence are shown in Supplementary Table S1). With every one-point increase in the score for domain 2 and domain 4, the risk of medication nonadherence increased by 19% (95% *CI*: 1.11–1.28) and 16% (95% *CI*: 1.06–1.27), respectively. With every one-point increase in the score for domain 8, the risk of medication nonadherence decreased by 30% (95% *CI*: 0.61–0.81). In addition, TB patients with a high education (*OR*_adj_ = 0.29; 95% *CI*: 0.08, 0.92) had a decreased risk of nonadherence compared with that in patients with a primary education or less, but the risk of nonadherence was higher for patients with a retirement salary [*OR*_adj_ = 2.55; 95% *CI* (1.16, 5.71)] than for those obtaining their living expenses from their children. Finally, the results of the Hosmer–Lemeshow goodness of fit test showed a *p*-value of 0.834 (>0.05), so the model had good reliability.

## 4 Discussion

Our main findings are that nearly half of the 405 elderly TB patients experienced moderate or high MRB. Among the eight domains of the LMQ-3, domain 8 had the highest median average score, followed by domain 4. Domain 3 had the lowest average score. Additionally, one-third of the elderly patients with TB had medication nonadherence, which was significantly associated with three domains (domains 2, 4, and 8). Moreover, both education level and source of living expenses are significant factors that affect medication adherence.

In our study, 49.4% and 42.7% of respondents perceived moderate and high MRB, respectively, which are different from those reported in New Zealand (Tordoff et al., 2019) and Bahrain (Alqallaf et al., 2022). In a study from New Zealand, moderate (45.1%) and high (30.5%) burdens were reported. This may be because our study included people aged ≥60 years, whereas in that study, it mainly included people aged ≥18 years, and those aged ≥65 years accounted for only 18.6%. A moderate burden (27.4%) and a high burden (72.2%) were reported in the Bahrain study. The high proportion of MRB in that study may be due to the inclusion of patients who had at least one chronic disease and used at least five prescription medications. However, only 19.3% of the patients in our study used at least five prescription medications. In addition, both the New Zealand and Bahrain studies involved people with chronic noncommunicable diseases, but our study involved people with TB. This may also be one of the reasons for the difference in MRB. Few studies are currently available for comparison to the present study since assessing MRB using the LMQ-3 among elderly TB patients is relatively new. This suggests that similar studies should be conducted in diverse regions to validate our findings, and longitudinal studies should be encouraged to assess how changes over time affect generalizability. However, comparisons between different studies should be considered with caution due to the differences in the demographic characteristics, health systems, environmental factors and so on.

Our study revealed that domain 8 and domain 4 were two main drivers of MRB. Domain 8 (control/autonomy of medicine use) had the highest median score, which is consistent with the results of a previous study (Tordoff et al., 2019). This may be because patients follow their doctors’ prescriptions too much and ignore their own control/autonomy of medicine use (Zhou et al., 2024). Domain 4 (side effects burden of prescribed medications) is a serious problem faced by TB patients (Khan et al., 2022). A prospective study showed that the incidence of side effects caused by TB drugs was 67.8%, and older people are more likely to experience side effects because of their weakened immunity (Choi et al., 2022). An unexpected finding in our study was that domain 3 (cost-related burden) had the lowest MRB. This may be related to Guizhou’s high medical insurance coverage since 2022 (National Bureau of Statistics of China, 2022), which improved reimbursement rates for TB outpatients (Guizhou Provincial People’s Government, 2022). Moreover, social protection policies provide subsidies for economically disadvantaged groups (National Bureau of Statistics, 2020).

In the present study, the incidence of medication nonadherence was 33.6% among elderly patients with TB, which is similar to that previously reported in Nigeria (30.5%) (Iweama et al., 2021) and India (31.3%) (Natarajan et al., 2022). Regarding the determinants of medication nonadherence, three domains (domains 2, 4, and 8) were significantly associated with medication adherence. First, our findings revealed that the greater the MRB of domain 2 (practical difficulties) perceived by patients, the higher their risk of nonadherence. This problem may be related to the decline in physical function and memory loss in elderly people. They may forget to take medicines (Chen et al., 2021; Gashu et al., 2021). Therefore, a variety of reminder methods, such as family members or smart tools, should be used to remind elderly people to take their medication. Investigating the practical difficulties of medication use among elderly TB patients is crucial for healthcare providers and policymakers, as it provides a reference for formulating intervention measures (Christopher et al., 2023). Second, domain 4 (side effect burden of prescribed medications) was a risk factor for treatment nonadherence in our study. This finding is consistent with previous studies (Chin et al., 2023; Xing et al., 2021). This finding may be due to side effects reducing patients’ quality of life (not only their health status but also their psychological and social wellbeing) (Ausi et al., 2021). Thus, it is necessary for doctors to assess, diagnose and treat side effects in a timely manner and provide psychological counseling for patients. Third, domain 8 (control/autonomy of medicine use) was a protective factor for medication adherence. The higher the domain 8 score is, the better the patients’ medication adherence. This may be because patients are more likely to strictly and passively follow doctors’ advice to take medicine (Zhou et al., 2024). Most respondents in our study reported that they would like to take medication as prescribed by the doctor. It is recommended that healthcare providers establish a doctor-patient relationship based on trust, cooperation, and respect. Specifically, they should pay attention to patients’ thoughts and concerns about their medication, spend more time listening to their feelings about the illness, and encourage patients to participate in prescription decisions, which helps enhance patients’ adherence with medication (Zhou et al., 2024).

This study also revealed that two demographic characteristics of patients (education level and source of living expenses) are associated with medication adherence. First, similar to other studies, patients with a high education level had better medication adherence than those with a primary education level (Lolong et al., 2023; Daba et al., 2020). Highly educated patients may become more knowledgeable about TB and may be more aware of harmful and serious nonadherence to TB, and be more motivated to manage their own health status (Jing et al., 2024; Ghassab-Abdollahi et al., 2024). This suggests that we should tailor health information services according to the educational levels of the elderly. Second, respondents whose living expenses came from retirement salaries were more likely to be nonadherent than those whose living expenses came from their children. Previous research has shown that family support plays a crucial role in promoting adherence (Chen et al., 2020) because family members provide financial, mental and emotional support to patients (Grigoryan et al., 2022; Nadon et al., 2023).

This study provides new insights into MRB and nonadherence among elderly TB patients in Guizhou. It contributes to the usefulness of LMQ-3 and allows important comparative work with future similar studies in countries with high TB burden and worldwide. Our findings have implications for TB control policies and specific recommendations for healthcare providers working in the field of TB healthcare. First, these findings provide evidence that LMQ-3 could be used in clinical practice and/or research, where assessing drug burden is important. This, in turn, could inform the necessity for personalized support and interventions. Second, it is recommended that health stakeholders develop multidisciplinary strategies, including the provision of tailored medication education programs and easily accessible information resources, as well as the development of policies and advocacy campaigns on prescribing. The interventions should also encourage fixed-dose drug combination to reduce MRB while promoting better adherence and clinical outcomes.

Our study has several limitations. First, the study sample was restricted to outpatients, so our results may not be representative of the MRB among inpatients and patients who were lost to follow-up. However, our study will spur future research to include a comprehensive sample and obtain more insight into MRB and adherence among elderly TB patients. Second, the cross-sectional study design is subject to recall bias and may not provide a strong ability to indicate causation. Third, our study lacks information on the duration of TB treatment for respondents. Future research should strive to obtain relevant information regarding treatment duration to further elucidate the association between MRB and medication adherence. Finally, the present study was conducted in Guizhou Province and may not represent the conditions in all of China. However, our findings may be helpful for other countries or regions with situations similar to those in Guizhou.

## 5 Conclusion

This study revealed that nearly half of the elderly TB patients in Guizhou Province suffered from moderate (49.4%) and high (42.7%) MRB, respectively. The results from eight domains of the LMQ-3 demonstrated that MRB in the study population was multidimensional. A total of 33.6% of patients were found to be nonadherent to their medications. Patients are more likely to be nonadherent if they experience high practical difficulties taking medicine and side effect burdens from prescribed medications. Patients also had an increased risk for nonadherence if they had low control/autonomy of medicine use. Our findings highlight that measuring MRB in multiple dimensions using the LMQ-3 in TB patients could assist clinicians in providing patient-centered treatment care plans to achieve optimal outcomes. Moreover, multifaceted interventions targeting the problems identified above should be implemented to reduce MRB and nonadherence among elderly TB patients in Guizhou. Our study emphasizes the value of continued research in this area despite the complexities involved in generalizing results. Understanding the extent to which these findings can be applied to other regions or countries is crucial for broader public health implications.

## Data Availability

The original contributions presented in the study are included in the article/Supplementary Material, further inquiries can be directed to the corresponding author.
