# Supplementary material for: Medication-related burden and its association with medication adherence among elderly tuberculosis patients in Guizhou, China: a cross-sectional study
Source: Front Pharmacol. 2024 Aug 29;15:1416005. doi: 10.3389/fphar.2024.1416005 (PMC11391241; doi:10.3389/fphar.2024.1416005)
Supplement: Supplementary file 1 [file DataSheet1.pdf]

Supplementary Material

1 Supplementary Figures and Tables

1.1 Supplementary Figures

1.1.1 Relationship between the eight domains and the logit-transformed values of medication adherence

There were linear relationships between each domain and the logit-transformed values of medication adherence shown in Supplementary Fig. 1.

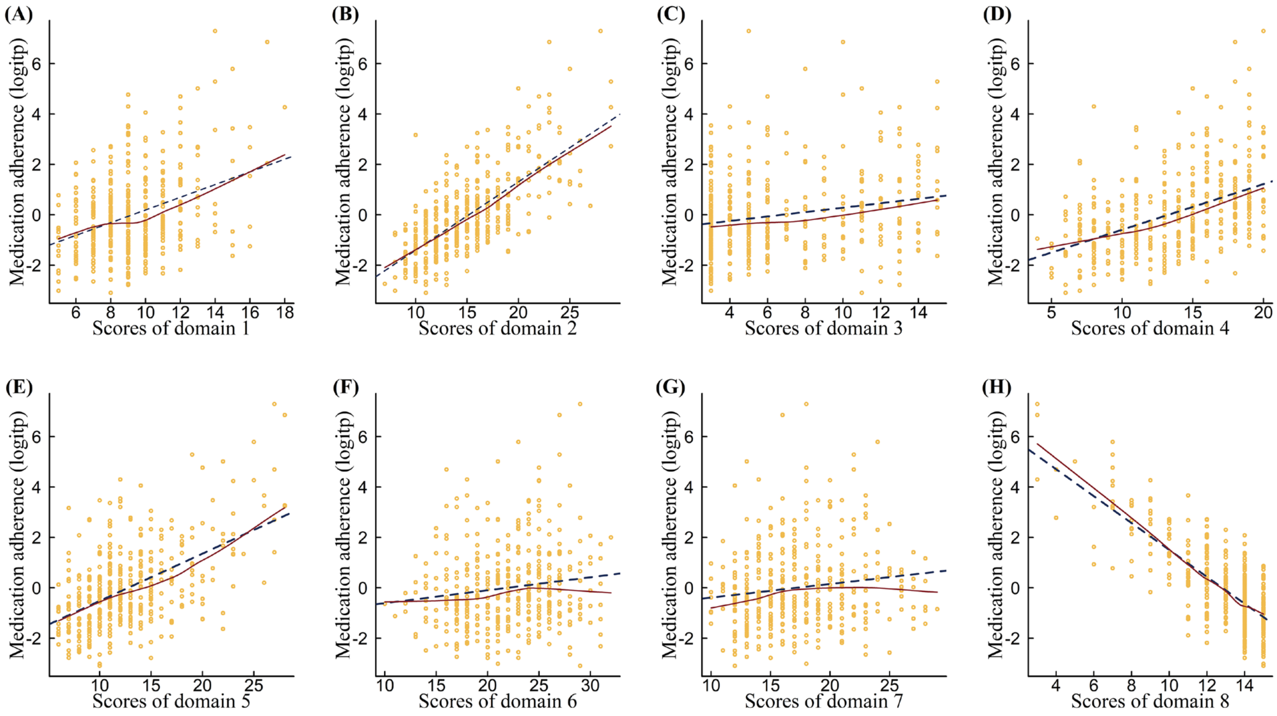

Supplementary Fig. 1 The component plus residual plot between the scores in eight domains and the logit-transformed values (logitp) of medication adherence.

1.1.2 The Pearson correlation between the scores in eight domains

All pearson correlation coefficient between the eight domains were less than 0.5, indicating that there was no siginificant multicollinearity relationship between them in Supplementary Appendix Fig. 2.

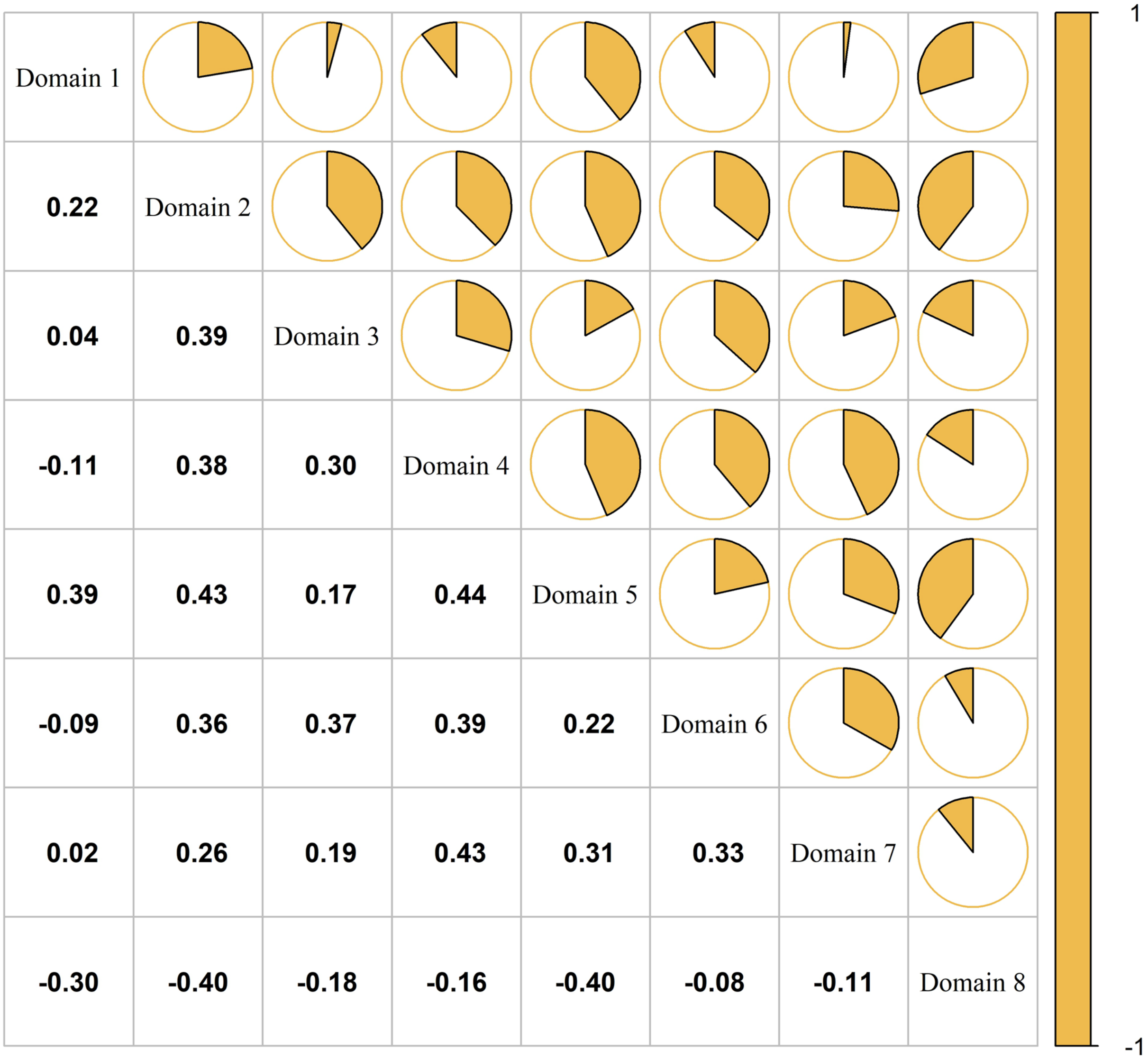

Supplementary Fig. 2. The Pearson correlation between the scores in eight domains.

1.2 Supplementary Tables

1.2.1 Associations between MRB and medication adherence after adjusting for covariates

Supplementary Table 1. Associations between MRB and medication adherence after adjusting for covariates.

Variables	Medication		OR_{crude} (95% *CI*)	OR_{adj} (95% *CI*)	*p*-value	*p*-value (LR-test)
	Adherence	Nonadherence				
**Eight domains Median (IQR)**						
Domain1	9.0 (8.0, 11.0)	9.0 (8.0, 12.0)	1.19 (1.09-1.29)	1.10 (0.97-1.26)	0.152	0.151
Domain2	14.0 (11.0, 16.0)	17.0 (14.0, 21.0)	1.24 (1.17-1.31)	1.19 (1.11-1.28)	< 0.001	<0.001
Domain3	5.0 (3.0, 9.0)	6.0 (3.0, 11.0)	1.07 (1.01-1.13)	0.95 (0.87-1.04)	0.212	0.209
Domain4	12.0 (9.0, 15.0)	16.0 (12.0, 18.0)	1.15 (1.09-1.21)	1.16 (1.06-1.27)	0.001	< 0.001
Domain5	11.0 (9.0, 14.0)	13.0 (10.0, 18.3)	1.14 (1.09-1.20)	0.98 (0.91-1.06)	0.714	0.714
Domain6	22.0 (18.0, 25.0)	23.0 (19.0, 26.0)	1.04 (0.99-1.09)	0.96 (0.89-1.03)	0.247	0.246
Domain7	16.0 (14.0, 19.0)	17.0 (14.8, 20.0)	1.04 (0.99-1.09)	0.94 (0.87-1.00)	0.078	0.073
Domain8	14.0 (13.0, 15.0)	12.0 (9.0, 14.0)	0.65 (0.58-0.73)	0.70 (0.61-0.81)	< 0.001	< 0.001
**Education level *n* (%)**						
Primary education or less	148 (62.7)	88 (37.3)	Ref.	Ref.		0.079
Secondary education	94 (69.6)	41 (30.4)	0.73 (0.47-1.15)	0.98 (0.53-1.79)	0.936	
High education	27 (79.4)	7 (20.6)	0.44 (0.18-1.04)	0.29 (0.08-0.92)	0.042	
**Source of living expenses *n* (%)**						
Children	151 (68.3)	70 (31.7)	Ref.	Ref.		0.043
Retirement salary	67 (65.1)	36 (35.0)	1.16 (0.71-1.90)	2.55 (1.16-5.71)	0.021	
Deposit	24 (75.0)	8 (25.0)	0.72 (0.31-1.68)	0.93 (0.32-2.51)	0.889	
Subsistence allowance	9 (40.9)	13 (59.1)	3.12 (1.27-7.63)	2.91 (0.94-9.24)	0.066	
Others	18 (66.7)	9 (33.3)	1.08 (0.46-2.52)	1.88 (0.63-5.19)	0.239	
**Loss productivity due to TB *n* (%)**						
Yes	27 (55.1)	22 (44.9)	Ref.	Ref.		0.093
No	242 (68.0)	114 (32.0)	0.58 (0.32-1.06)	0.51 (0.24-1.11)	0.091	
**Treatment classification of TB *n* (%)**						
Initial treatment	215 (69.4)	95 (30.6)	Ref.	Ref.		0.467
Retreatment	54 (56.8)	41 (43.2)	1.72 (1.07-2.76)	1.25 (0.69-2.25)	0.465	
**ADRs *n* (%)**						
Yes	203 (63.0)	119 (37.0)	Ref.	Ref.		0.695
No	66 (79.5)	17 (20.5)	0.44 (0.25-0.78)	1.17 (0.56-2.54)	0.694	

Abbreviations: MRB, medication-related bueden; OR_{crude}, cruded odds ratio; OR_{adj}, adjusted odds ratio; *CI*, confidence interval; Ref., reference group; TB, tuberculosis; ADRs, adverse drug reactions
